# Long-term results of etiology-based thoracic endovascular aortic repair: a single-center experience

**DOI:** 10.1007/s00380-024-02392-8

**Published:** 2024-04-09

**Authors:** Akitoshi Takazawa, Toshihisa Asakura, Ken Nakazawa, Osamu Kinoshita, Hiroyuki Nakajima, Akihiro Yoshitake

**Affiliations:** 1 Department of Cardiovascular Surgery, Saitama Medical University International Medical Center, Yamane, Hidaka City, Saitama 1397-1 Japan; 2 Department of Radiology, Saitama Medical University International Medical Center, Hidaka City, Japan

**Keywords:** Thoracic endovascular aortic repair, Thoracic aortic aneurysm, Stanford type B aortic dissection, Etiology, Long-term outcomes

## Abstract

The use of thoracic endovascular aortic repair (TEVAR) for thoracic aortic aneurysm (TAA) and Stanford type B aortic dissection (TBAD) has been increasing; however, in terms of etiology, the differences of long term after TEVAR outcomes remain unexplored. Thus, we investigated etiology-specific long-term results of TEVAR for TAA and TBAD. A total of 421 TEVAR procedures were performed at our institution from July 2007 to December 2021; 249 TAA cases and 172 TBAD cases were included. Traumatic aortic dissection and aortic injury cases were excluded. The mean observation duration was 5.7 years. The overall 30-day mortality rate was 1.4% (*n* = 6), with 1.2% (*n* = 3) in the TAA group and 1.7% (*n* = 3) in the TBAD group. The overall incidence of postoperative stroke was 0.9% (*n* = 4), with 1.2% (*n* = 3) and 0.6% (*n* = 1) in the TAA and TBAD groups, respectively (*p* = 0.90). Paraplegia developed in 1.7% (*n* = 7) of patients, with 2.4% (*n* = 6) in the TAA group and 0.6% (*n* = 1) in the TBAD group. Freedom from aortic-related death was not significantly different between the two etiologies; however, thoracic reintervention was more common in the TBAD group (*p* = 0.003), with endoleak being the most common indication for reintervention. Additionally, retrograde type A aortic dissection occurred in four TBAD cases, while migration occurred in three TAA cases. The perioperative results of TEVAR for TAA and TBAD were satisfactory. The long-term results were unfavorable owing to the occurrence of etiology-specific and common complications. In terms of the high frequency of reintervention, the long-term complications associated with TEVAR are etiology specific.

## Introduction

Thoracic endovascular aortic repair (TEVAR) has become the main strategy for the treatment of various thoracic aortic pathologies of multiple etiologies [1]. During the past decade, endovascular therapy has revolutionized the management of descending thoracic aortic aneurysm (TAA) with the benefits of preventing an aortic rupture without the need for direct surgical exposure. However, the long-term durability of TEVAR devices has recently become a cause for concern. Furthermore, a different strategy is required for the treatment of TAA and type B aortic dissection (TBAD), potentially resulting in varying outcomes. The purpose of this single-center study was to review the long-term results of TEVAR treatment performed at our institution for different etiologies of TAA and TBAD.

## Materials and methods

### Study design and population

The data were retrospectively obtained from a storage/software facility at Saitama Medical University International Medical Center. We reviewed the data of consecutive patients who underwent TEVAR for TAA and TBAD at our center between July 2007 and December 2021. We excluded patients who underwent TEVAR for blunt thoracic aortic injury. All these patients had a high surgical risk due to comorbidities, such as chronic obstructive pulmonary disease, coronary artery disease, and renal insufficiency. Moreover, patients were selected in cases where they were both technically and anatomically suitable for TEVAR. Eligible patients were divided into two groups according to their etiology: group A (TAA) and group B (TBAD). Early-, mid-, and long-term results were analyzed and compared between the groups.

A total of 421 patients were treated by TEVAR, 249 for TAA (group A), and 172 for TBAD (group B). The mean follow-up period was 68.8 ± 39.5 (range, 0.0–157.8) months. In group B, the average duration between onset and operation was 579.4 days, which was 20 in acute phase, 30 in subacute, and 122 in chronic. The study protocol was approved by the Institutional Review Board of Saitama Medical University International Medical Center, where the work was conducted (ID: 2022–116; December 7, 2022).

### Procedures and treatment

All procedures were performed under general anesthesia. The femoral artery or external iliac artery was incised. The stent graft was deployed under rapid pacing (heart rate > 150 beats/min). In cases of arch lesions, an extra-anatomical bypass of neck vessels was performed to secure the landing zone. In group A, TEVAR was performed to interrupt blood flow in the aneurysm. In group B, TEVAR was performed to exclude the primary entry. After accessing the true lumen, a stiff guidewire was then positioned in the ascending aorta, and an endograft was deployed. A pigtail catheter was introduced into the ascending aorta through the contralateral femoral or brachial artery, and digital subtraction angiography was performed. Touchup ballooning of the endograft was avoided. The device oversizing was kept at < 10% compared to the native aorta.

### Endpoints

The primary endpoints were 30-day mortality after TEVAR and early and late aortic-related deaths. Late aortic-related deaths were defined as those that occurred more than 30 days after the initial procedure. The cause of aortic-related death was obtained from medical records, telephone investigations, or autopsy reports. The secondary endpoint was aortic reintervention, which was defined as the need to perform an additional procedure to resolve a complication resulting from the initial TEVAR.

Technical success was defined based on the Society of Vascular Surgery reporting standards on perioperative events within 24 h postoperatively [2].

### Study variables and definitions

TBAD was defined according to the Stanford classification, that is, dissection of the entry site distal to the left subclavian artery. The diagnosis was based on clinical history and non-invasive, diagnostic computed tomography (CT) angiography.

Adverse events of early outcomes were defined as hospital death, stroke, spinal cord injury, and respiratory failure requiring tracheostomy. Hospital death was defined as death between hospital admission and discharge or within 30 days postoperatively. Stroke was diagnosed using CT or magnetic resonance imaging in case of a new occurrence of postoperative neurological symptoms. Paraplegia was defined as a permanent bilateral motor deficit in the lower extremities.

Adverse events of mid- and long-term outcomes included aortic-related death, retrograde type A aortic dissection (RTAD), aorto-esophageal fistula (AEF), and aorto-bronchial fistula (ABF). Thoracic aortic reintervention was defined as additional open or endovascular repair of the descending thoracic and thoracoabdominal aorta due to aortic disease progression.

### Statistical analysis

Categorical variables are expressed as numbers (percentage of total), while continuous variables are presented as mean ± standard deviation. The *χ*^2^ test was used to compare categorical variables. Continuous variables were compared using Student’s *t*-test. The cumulative rate was determined using the Kaplan–Meier method. Logistic regression analysis was used to examine the significance of the clinical, diameter-calculated CT scan, and operative variables. Differences in outcomes were considered statistically significant at *p* < 0.05. All statistical analyses were performed using JMP, version 14.0 (SAS Institute Inc., Cary, NC, USA).

## Results

The patients’ baseline characteristics are summarized in Table 1. None of the participants in both groups had missing data for any variables. The two groups (group A and group B) exhibited significant differences in terms of age (66.3 ± 11.1 vs 74.0 ± 8.3, *p* < 0.001), diabetes mellitus (8.7% vs 24.1%, *p* < 0.001), chronic kidney disease (19.8% vs 30.9%, *p* = 0.01), coronary artery disease (16.9% vs 35.3%, *p* < 0.01), chronic obstructive pulmonary disease (22.8% vs 41.8%, *p* < 0.001), peripheral artery disease (5.8% vs 41.8%, *p* = 0.01), and antiplatelet or anticoagulation therapy (34.3% vs 46.6%, *p* = 0.01). There were no significant differences between the two groups for other comorbidities.

**Table 1:** Patient characteristics

Variables	group A (*N* = 249)	group B (*N* = 172)	*p*-value
Age (years)	74.0 ± 8.3	66.3 ± 11.1	< 0.001
Male	192 (77.0)	132 (76.7)	0.99
Hypertension	211 (84.7)	163 (94.8)	0.4
Dyslipidemia	105 (42.2)	75 (43.1)	0.87
Diabetes mellitus	60 (24.1)	15 (8.7)	< 0.001
Chronic kidney disease	77 (30.9)	34 (19.8)	0.01
Coronary artery disease	88 (35.3)	29 (16.9)	< 0.001
Chronic obstructive pulmonary disease	104 (41.8)	39 (22.8)	< 0.001
Peripheral artery disease	35 (14.1)	10 (5.8)	0.01
Antiplatelet or anticoagulation therapy	116 (46.6)	59 (34.3)	0.01

Data are presented as mean ± standard deviation or *n* (%)

Operative details are presented in Table 2. In group A, the surgery was elective in 241 patients (80.7%) and emergent in 48 (19.3%; rupture, 34; impending rupture, 14), and in group B, it was elective in 138 (80.2%) and emergent in 34 (19.8%; rupture, 17; impending rupture, 14; malperfusion, 3). One-debranching TEVAR (right axillary to left axillary artery bypass) was required in 8 (3.2%) patients in group A and 10 (5.8%) patients in group B. Two-debranching TEVAR (right axillary artery to left carotid artery and left axillary artery bypass) was performed in 8 (3.2%) patients in group A and 4 (2.3%) in group B.

**Table 2:** Operative status and procedure

Variables	group A (*N* = 249)	group B (*N* = 172)	*p*-value
Operative status			
Elective	201 (80.7)	131 (80.2)	0.9
Emergent	48 (19.3)	34 (19.8)	
Rupture	48 (19.3)	31 (18.0)	
Malperfusion	–	3 (1.8)	
Operative procedure			
1 debranching	8 (3.2)	10 (5.8)	0.19
2 debranching	8 (3.2)	4 (2.3)	0.81
Total debranching	2 (0.8)	0 (0.0)	0.24
Proximal landing zone			
Zone 0	2 (0.8)	0 (0.0)	0.24
Zone 1	8 (3.2)	4 (2.3)	0.81
Zone 2	74 (29.7)	50 (29.1)	0.89
Zone 3	79 (31.7)	61 (35.5)	0.42
Zone 4	63 (25.3)	46 (26.7)	0.74
Zone 5	23 (9.2)	11 (6.4)	0.29

Data are presented as *n* (%)

### Perioperative and early complications

Perioperative outcomes are summarized in Table 3. One patient from each groups (A and B) died intraoperatively: in group A, due to cardiac tamponade due to RTAD, and in group B, due to intraoperative aortic rupture in one case of acute, complicated TBAD. The overall 30-day mortality was 1.4% (*n* = 6), 1.2% (*n* = 3) in group A and 1.7% (*n* = 3) in group B. The overall incidence of postoperative stroke was 0.9% (*n* = 4), 1.2% (*n* = 3) in group A and 0.6% (*n* = 1) in group B, although no significant difference was observed between the groups (*p* = 0.90). Additionally, 1.7% (*n* = 7) of patients developed spinal cord injury, 2.4% (*n* = 6) in group A and 0.6% (*n* = 1) in group B, with no significant difference (*p* = 0.15). One patient in group A had an embolism of the superior mesenteric artery within the shaggy aorta and died of intestinal necrosis 2.6 months postoperatively. Respiratory failure requiring tracheostomy was not observed in any patient.

**Table 3:** Early results

Variables	group A (*N* = 249)	group B (*N* = 172)	*p*-value
30-day mortality	3 (1.2)	3 (1.7)	0.96
Stroke	3 (1.2)	1 (0.6)	0.9
Paraplegia	6 (2.4)	1 (0.6)	0.15
Pneumonia	0 (0.0)	1 (0.6)	0.23
Mesenteric arterial embolism	1 (0.4)	0 (0.0)	0.41
Renal failure	3 (1.2)	3 (1.7)	0.96

Data are presented as n (%)

### Mid- and long-term outcomes

**Fig. 1:**
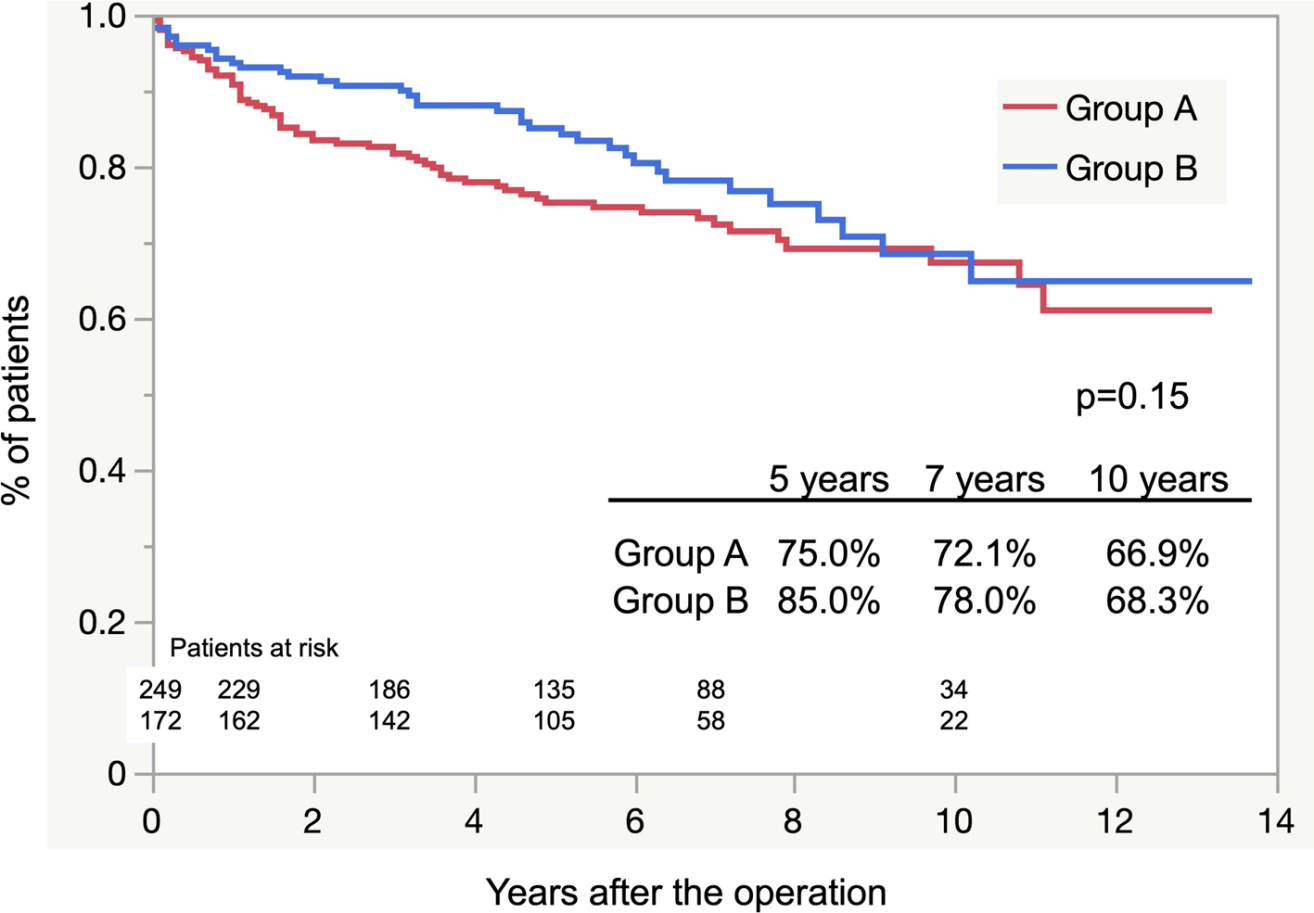
Freedom from all causes of death curves

**Fig. 2:**
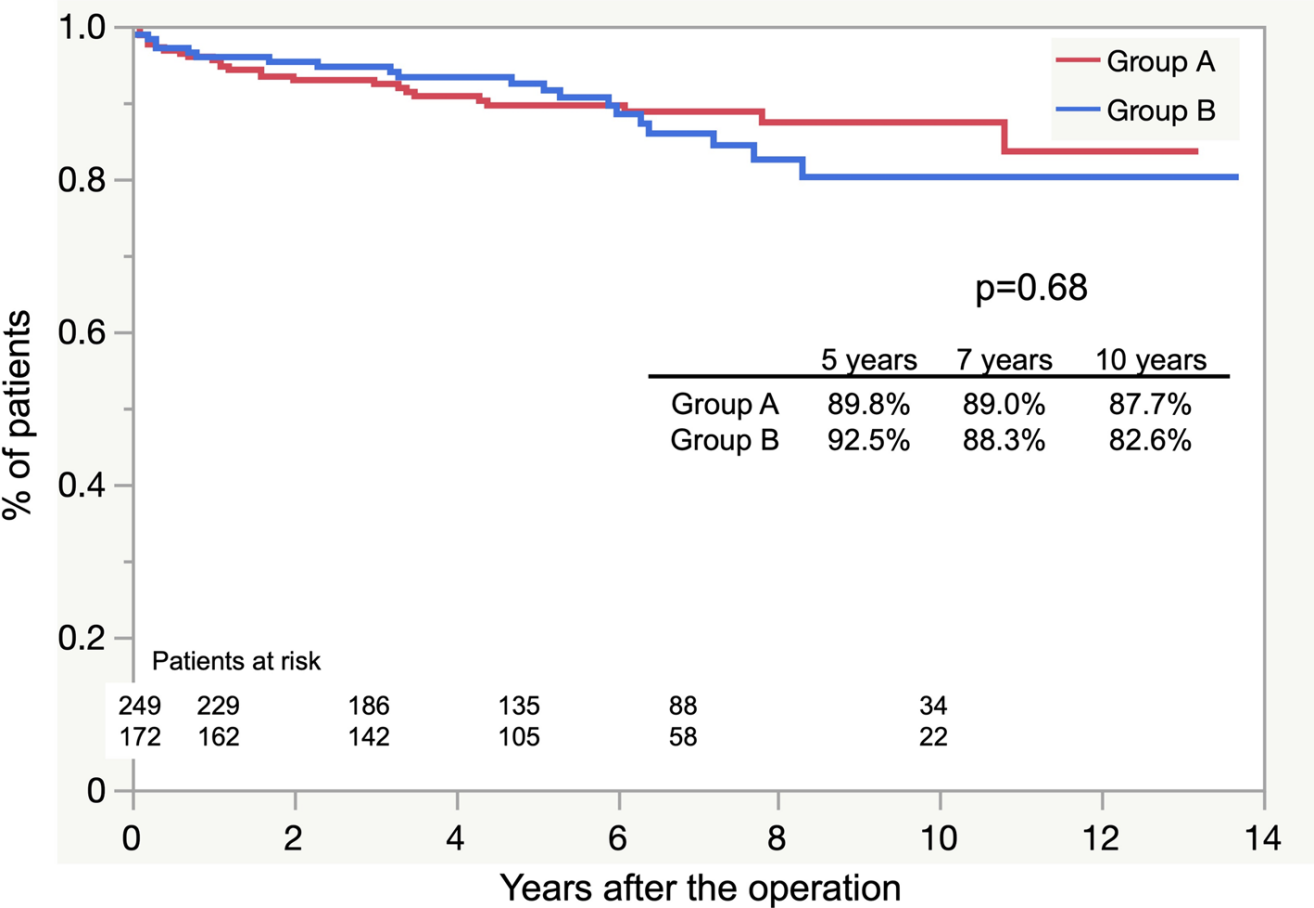
Freedom from aortic-related death curves

The overall estimated postoperative survival rates at 3, 5, 7, and 10 years did not differ significantly between both groups (*p* = 0.15; Fig. 1). Freedom from aortic-related death at 5, 7, and 10 years also showed no significant difference (*p* = 0.60; Fig. 2). The causes of aortic-related death were rupture (*n* = 10), infection (*n* = 3), AEF (*n* = 4), ABF (*n* = 4), and RTAD (*n* = 1). The cause of death was not confirmed in 20 patients. Non-aorta-related deaths due to malignancy were more prevalent in group A than in group B, with a significant difference (*p* = 0.01). The details of all causes of death for each group are presented in Table 4.

**Table 4:** All causes of death

Variables	group A (*N* = 249)	group B (*N* = 172)	p-value
Aorta-related death			
Rupture	5 (2.0)	5 (2.9)	0.78
Infection	1 (0.4)	2 (1.2)	0.74
Aorto-bronchial fistula	3 (1.2)	1 (0.6)	0.89
Aorto-esophageal fistula	1 (0.4)	3 (1.7)	0.38
Retrograde type A aortic dissection	0 (0.0)	1 (0.6)	0.85
Myocardial ischemia	2 (0.8)	0 (0.0)	0.65
Heart failure	2 (0.8)	3 (1.7)	0.68
Stroke	4 (1.7)	6 (3.5)	0.36
Pneumonia	10 (4.0)	5 (2.9)	0.74
Renal failure	2 (0.8)	3 (1.7)	0.68
Intestinal hemorrhage	4 (1.7)	1 (0.6)	0.62
Malignancy	14 (5.6)	1 (0.6)	0.01
Unknown	13 (5.2)	7 (4.0)	0.56

Data are presented as *n* (%)

**Fig. 3:**
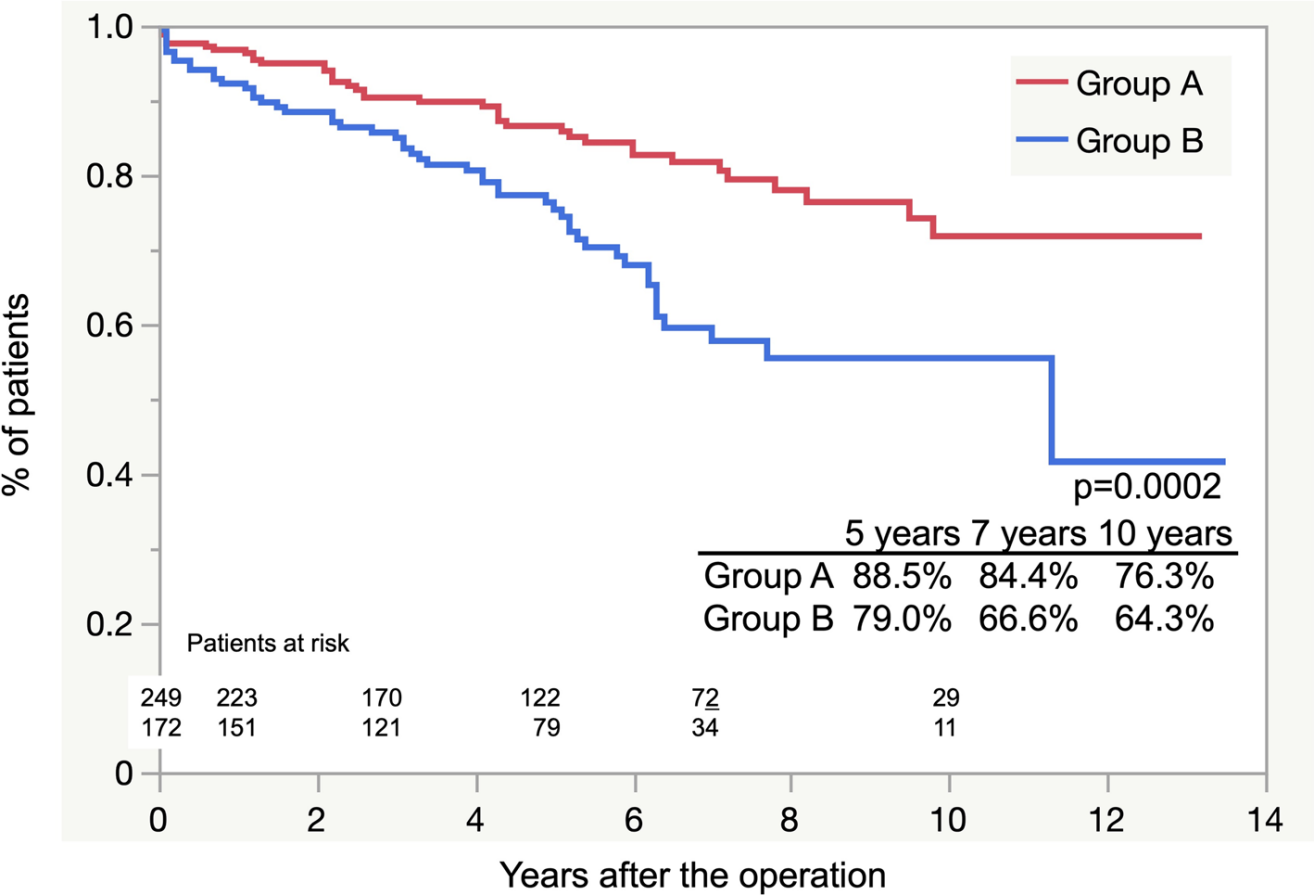
Freedom from thoracic reintervention curves

Figure 3 shows the estimated Kaplan–Meier curves of freedom from thoracic reintervention for the two groups. The overall postoperative freedom from thoracic reintervention at 5, 7, and 10 years was 88.5 ± 2.9%, 84.4 ± 4.8%, and 76.3 ± 8.8%, respectively, in group A, and 79.0 ± 3.8%, 66.6 ± 5.9%, and 64.3 ± 6.3%, respectively, in group B (*p* = 0.003). The indications for reintervention are shown in Table 5; in both groups, endoleak was the most common reason (18 and 13 in group A and group B, respectively), and sac enlargement was more prevalent in group B than in group A, with a significant difference (6 and 14 in groups A and B, respectively; *p* = 0.007). Four patients in group B also had RTAD. Otherwise, migration was noted in three patients in group A, two of whom underwent open surgery (thoracoabdominal aortic replacement and descending aorta replacement) and later died. In group B, six patients required additional procedures due to the sac enlargement by the residual re-entry (four patients underwent additional TEVAR and two underwent Candy Plug false lumen embolization). One patient who underwent thoracoabdominal aorta replacement died intraoperatively, and one patient had an infection with newly developed ABF, leading to late death. In group B, no deaths occurred in patients who underwent open surgery.

**Table 5:** Causes of thoracic reintervention

Variables	group A (*N* = 249)	group B (*N* = 172)	*p*-value
Sac enlargement	6 (2.4)	14 (8.1)	0.007
Residual re-entry flow	0 (0.0)	6 (3.5)	0.01
Unknown	6 (2.4)	8 (4.6)	0.32
Migration	3 (1.2)	0 (0.0)	0.39
Endoleak	18 (7.2)	13 (7.6)	0.95
type 1a	4 (1.6)	4 (2.3)	0.87
type 1b	3 (1.2)	2 (1.2)	0.97
type 2	4 (1.6)	1 (0.6)	0.62
type 3	4 (1.6)	6 (3.5)	0.36
type 4	3 (1.2)	0 (0.0)	0.15
Aorto-bronchial fistula	1 (0.4)	0 (0.0)	0.40
Aorto-esophageal fistula	0 (0.0)	1 (0.6)	0.23
Retrograde type A aortic dissection	0 (0.0)	4 (2.3)	0.02
Stent graft-induced new entry	5 (2.0)	9 (5.2)	0.12

Data are presented as *n* (%)

## Discussion

In this study, the short-term results of TEVAR for TAA and TBAD were satisfactory, whereas the long-term results of both groups were unfavorable due to the occurrence of etiology-specific and common complications. We found no significant differences in all-cause mortality and aortic-related death between the two pathologies.

The current standard of care for a descending thoracic aortic lesion (aneurysm, blunt traumatic aortic injury, and type B dissection) is TEVAR, which is preferentially recommended over surgery if the pathology meets specific anatomical requirements [3–8]. The main objective of TEVAR in TAA is to treat and prevent aneurysm rupture and decompression, while in TBAD, it is to decrease false lumen blood flow and increase true lumen blood flow by closing the entry site and prevent false lumen expansion and rupture by a false lumen thrombus.

The long-term benefits of TEVAR for TAAs remain unclear. In our study, the rate of long-term freedom from aortic-related death after TEVAR was relatively high (87.7%); however, some patients developed complications of endoleaks (7.6%) after TEVAR, which led to aortic-related death due to rupture (2.0%) and necessitated reintervention, including late open conversion. In TEVAR for TAAs, when the proximal landing zone is somewhat limited and the aortic aneurysm itself does not remodel, stent grafts tend to migrate distally. Ranney reported a similar excellent long-term (12-year) aorta-specific survival rate after TEVAR (96.2%) and noted that reintervention due to endoleaks occurred in 7% of cases [9].

The gold standard treatment for acute and chronic TBAD remains optimal medical therapy. This is aimed at limiting the progression of dissection by reducing aortic wall pressure. However, whether conservative therapy is effective for TBAD is unclear. In particular, chronic aortic dissection carries a high risk of late aneurysmal dilation, mainly due to false lumen enlargement and rupture. In these cases, operative treatment is indicated. For chronic TBAD, the preference for endovascular surgery remains controversial. However, endovascular repair does not appear to deliver the expected results. In this study, two patients who underwent TEVAR for TBAD died due to RTAD, and four patients were operated emergently. The choice of TEVAR for TBAD remains controversial because stent grafting bears the risk of eliminating antegrade false lumen flow by persisting through the primary entry, while retrograde false lumen flow persists through potential dissection re-entry more distally. The frequent need for reintervention to address specific complications like RTAD and SINE, which are caused by intimal damage and residual re-entry, may not completely prevent rupture due to false lumen enlargement. Guangqi et al. reported their experience with 121 consecutive patients who underwent endovascular repair for acute and chronic TBAD. They found that postoperative endoleaks occurred in 22% of cases, with a 30-day mortality rate of 8.2% [10]. TEVAR is associated with a high 30-day mortality rate and severe complications, including RTAD (2.5–8%) [11, 12], stroke (4.6%), and paraplegia (1.9–4.4%) [13, 14]. Furthermore, there were a small number of fatal complications, such as AEF and ABF, during the mid- and long-term observation periods. In our study, the causes of aorta-related deaths in group B included rupture in five cases, infection in two cases, AEFs in three cases, ABFs in one case, and RTAD in one case. Performing TEVAR in cases with a considerably enlarged false lumen may carry a high risk of fistula formation. Nozdrzykowski et al. indicated that patients with chronic TBAD with extensive aneurysms, malperfusion, or acute rupture are surgically challenging, and the use of TEVAR might be limited due to the aneurysm size and location, occlusion by dissection of the false lumen, or thrombus formation within the chronic aneurysm [15]. TEVAR for TBAD has been used for treatment up to the celiac artery and not up to the abdominal aorta, including the abdominal visceral branches that arise partially or totally from the false lumen. Gao et al. illustrated that the maximum abdominal aorta diameter and the number of branches arising from the false lumen were independent risk factors for incomplete false lumen thrombosis in the subacute phase [16].

Several studies have examined the long-term results for each etiology, including ours. Our findings revealed no difference in all-cause mortality and aortic death between the two pathologies, although TBAD required more secondary treatments than TAAs in the long term. With additional reintervention at the appropriate time and indications, TBAD may also safely enhance long-term survival.

However, patients’ characteristics differed significantly between the two groups. Especially, the death of patients in group A mainly resulted from non-aorta-related causes, such as cardiac failure, pneumonia, and malignancy. Some reports have indicated that long-term outcomes depend on the aortic pathology and patients’ comorbidities, and a TAA is the most complicated pathology and causes the highest mortality [17–19]. Previous series of patients undergoing treatment indicated that most deaths after TEVAR for TAAs were due to cardiac failure, pneumonia, and cancer [20, 21]. On the other hand, the causes of death in patients in group B remained largely unknown, especially in terms of aorta-related deaths. A similar study reported that some important elements, such as the influence of medical risk factor control on mid-term results and accurately recording the cause of death, could not be studied, which may lead to an underestimation of the deaths [22]. We considered that the presence of different factors in the two groups may have influenced the long-term results.

### Limitations

Our study had the following limitations. First, it was a retrospective study with possibly unavoidable patient selection bias; this may lead to partially improved results compared to real-life anatomical and clinical scenarios. Second, it included a small sample size. Third, the rate of major adverse outcomes was probably underestimated in both pathologies; this might have led to biased analysis results. Fourth, we did not classify the onset into acute, subacute, or chronic phases. In the present study, the outcome of TBAD may vary depending on the onset and phases, as it is influenced by the achievement of aortic remodeling.

### Conclusions

The early outcomes of TEVAR for both TAA and TBAD were satisfactory. However, the mid- and long-term results could not be assessed owing to the occurrence of etiology-specific and other common complications. The purpose of TAA and TBAD is not the same; therefore, the main adverse events are different. The occurrence rates of migration and paraplegia are higher in TAA. On the other hand, TBAD remains a challenging etiology that should be considered when assessing reintervention rate. Long-term complications associated with TEVAR are etiology specific.

## Data Availability

The datasets generated and analyzed during the current study are available from the corresponding author on reasonable request.
